# Barriers and Facilitators of Conducting Medication Reviews in Nursing Home Residents: A Qualitative Study

**DOI:** 10.3389/fphar.2019.01026

**Published:** 2019-09-18

**Authors:** Hans Wouters, Juliet M. Foster, Anne Ensink, Lisa Kouladjian O’Donnell, Sytse U. Zuidema, Froukje Boersma, Katja Taxis

**Affiliations:** ^1^Department of PharmacoTherapy, Epidemiology & Economics (PTEE), Faculty of Science and Engineering, Groningen Research Institute of Pharmacy, University of Groningen, Groningen, Netherlands; ^2^Department of General Practice and Elderly Care Medicine, University Medical Center Groningen, University of Groningen, Groningen, Netherlands; ^3^Woolcock Institute of Medical Research, University of Sydney, Sydney, NSW, Australia; ^4^NHMRC Cognitive Partnership Centre, University of Sydney, Sydney, NSW, Australia; ^5^Department of Clinical Pharmacology and Aged Care, Royal North Shore Hospital, University of Sydney, Sydney, NSW, Australia

**Keywords:** polypharmacy, inappropriate prescribing, semi-structured interviews, deprescribing, geriatrics, qualitative study, inter-professional collaboration, medication reviews

## Abstract

**Objectives:** Inappropriate medication prescribing is a recognized clinical problem in nursing home residents of whom many have polypharmacy. However, results about the effectiveness of medication reviews targeted at improving prescribing and deprescribing have been equivocal. We therefore examined barriers and facilitators of conducting medication reviews.

**Method:** We purposively sampled medication reviews to capture salient barriers and facilitators of conducting medication reviews both in nursing home care units for dementia and disabling conditions. We held semi-structured interviews about consecutive steps of medication reviews. Interviews were transcribed verbatim and analyzed with the “method of constant comparison.”

**Results:** Six nursing home residents/relatives of nursing home residents, 8 elder care physicians, 5 pharmacists, and 10 nurses took part in the semi-structured interviews. We observed four overarching themes of barriers and facilitators: “realizing fidelity of the patient perspective (theme 1),” “level of comprehensiveness of medication reviews (theme 2),” “inclinations of healthcare providers (theme 3),” and “inter-professional collaboration and alliances (theme 4).” Theme 1 “realizing fidelity of the patient perspective” referred to the observation that assessing the patient perspective was a delicate balance between the value and the impediments of a proper assessment of the patient perspective. Theme 2 “level of comprehensiveness of medication reviews” reflected the struggle of practitioners to find an optimum between medication reviews being both comprehensive and feasible. Theme 3 “inclinations of healthcare providers” concerned setting intervention targets that were complementary to the practices of physicians and keeping the pharmacist blind to the patient perspective as a countermeasure to physicians’ inclinations. Finally, theme 4 “inter-professional collaboration and alliances” highlighted mutual support and inter-professional collaboration to strengthen healthcare practitioners’ contributions.

**Discussion:** These themes of barriers and facilitators emphasize the need to improve meta-communication during the medication review process. This pertains to the need for healthcare providers to appraise the fidelity of the patient perspective in a dialogue with residents/relatives. Furthermore, discourse between healthcare practitioners is needed beforehand about the level of comprehensiveness intervention targets, and inter-professional collaboration.

## Introduction

Inappropriate medication prescribing is a recognized clinical problem in nursing home residents of whom many have polypharmacy. Up to 40% of nursing home residents have ≥1 inappropriate medications prescribed (Gallagher et al., 2007). Medication reviews have been examined as an intervention strategy to improve prescribing, (Holland et al., 2005; Zermansky et al., 2006; Gillespie et al., 2009; Patterson et al., 2010; Frankenthal et al., 2014; Wallerstedt et al., 2014; Meid et al., 2015) but findings about their effectiveness have been equivocal (Alldred et al., 2016). Recently, we conducted the Discontinuing Inappropriate Medication in Nursing Home Residents (DIM-NHR) study, a pragmatic cluster randomized controlled trial (RCT) (Wouters et al., 2017). The aim of the DIM-NHR study was to examine successful discontinuation of inappropriate medication use and to improve prescribing in nursing home residents with Multidisciplinary Multistep Medication Reviews (3MR). 3MR medication reviews were carried out by elder care physicians in collaboration with pharmacists and nursing staff. 3MR medication reviews involved an assessment of the patient perspective and medical information, drug reviewing, a multidisciplinary meeting by elder care physicians and pharmacists to arrive at pharmacotherapeutic actions, and the actual execution of these actions. Results demonstrated that 3MR medication reviews were effective in discontinuing inappropriate medication use in nursing home residents.

Medication reviews, such as the 3MR, can be considered complex healthcare interventions because they consist of consecutive steps and are usually conducted by an inter-professional team (Craig et al., 2008). As such, complex healthcare interventions are difficult to evaluate with solely quantitative methods, hence qualitative research may be worthwhile to conduct. Qualitative analysis is likely to shed light on the barriers and facilitators of consecutive steps of medication reviews (2016). Insights from qualitative research into challenges have the potential to improve the success rate of medication reviews in nursing homes as well as other relevant care settings for older geriatric patients. Inadequate dealing with different barriers and not optimally using facilitators is likely to have caused the results of previous studies to be equivocal. Barriers and facilitators are expected to occur with regard to two central components of the medication review process, namely, patient involvement and inter-professional collaboration. Patient involvement is important to provide healthcare according to the ethical principles of “beneficence,” “non-maleficence,” “patients’ autonomy,” and “justice” (Reeve et al., 2016). However, more knowledge is needed about barriers and facilitators of incorporating the patient perspective and shared decision-making as part of medication reviews. It has not yet been widely examined how and to what extent nursing home residents, of whom many have dementia and cognitive impairment, could be involved in decision-making about prescribing (Reeve et al., 2016). Furthermore, regarding inter-professional collaboration, there is a paucity of knowledge about the mutual perceptions of physicians, pharmacists, and nursing staff about each other´s roles and duties in conducting the consecutive steps of medication reviews, and in deciding on and executing of changes. In addition to the previously published quantitative evaluation (Wouters et al., 2017), we therefore also embedded a qualitative study in the DIM-NHR cluster RCT.

Accordingly, the aim of this qualitative study was to examine the barriers and facilitators of conducting medication reviews aimed at improving medication prescribing in nursing home residents.

## Materials and Methods

### Qualitative Study

Between September 2014 and January 2016, we conducted a qualitative study following established methods (Miles and Huberman, 1994) and the “Consolidated Criteria for Reporting Qualitative Research” (COREQ) recommendations (Consolidated criteria for reporting qualitative research (COREQ), 2016). We utilized in-depth, semi-structured interviews to research the medication review experiences of nursing home residents (or their representative) and their physician, pharmacist, and nursing staff. Interviewees were selected from DIM-NHR study participants. Nursing home residents were included in the DIM-NHR study if they had a life expectancy that was greater than 4 weeks. Elder care physicians were eligible for entry into the DIM-NHR study if they were responsible for dementia special care units or care units for disabling conditions. Details of the DIM-NHR study have been described elsewhere (Wouters et al., 2017).

We adopted purposive sampling to uncover salient problems that were likely to be encountered while conducting medication reviews. We therefore selected the medication reviews conducted for nine nursing home residents that reflected diversity in terms of the nursing home residents’ sex, reason for admission (dementia or physical disability), and number and type of prescribed medications. We approached the treating elder care physician and the pharmacist involved in the medication review, a member of the nursing staff, and the selected nursing home resident (or their representative, usually a family member, in cases were the nursing home resident was incapacitated). Nursing home residents were interviewed in their own room (or their representative in their own home). Healthcare professionals were interviewed individually in their own offices.

### Data Generation

All interviews were conducted by HW, a male researcher with experience in conducting qualitative research, with knowledge of polypharmacy, medical decision-making, and patients’ attitudes toward medication use, geriatrics and gerontology, and cognitive function. Most of the participants had met HW at least once prior to the interview and were aware that he was one of the DIM-NHR study trialists. Interviews were expected to last 1 h and were audio recorded. Field notes were also made.

### Interview Guide of Semi-Structured Interviews

The interview guide was developed during multiple rounds of discussion between HW, FB, and KT. The interviews concerned the four consecutive steps of the 3MR (see **Table 1**): “assessing patient perspective and medical information (step 1),” “drug reviewing (step 2),” “multidisciplinary meeting and pharmacotherapeutic actions (step 3),” and “execution and evaluation of pharmacotherapeutic actions (step 4).” Step 1 reflected the patient involvement in medication reviews. Steps 2–4 reflected inter-professional collaboration by physicians, pharmacists, and nursing staff. Each interview ended with a reflection on issues discussed and potential omissions. To identify both expected and unexpected barriers and facilitators of conducting medication reviews as specifically as possible, healthcare providers were prompted at the beginning of the interview by presenting them the medication review that had been selected by the research team. To that end, HW reminded healthcare providers about the specific pharmacotherapeutic problems that had been encountered during the medication review.

**Table 1:** Interview questions about the four consecutive medication review steps.

	Steps and key questions
#1	*Assessing patient perspective and medical information* *To physicians and pharmacists:* Was it possible to assess this nursing home resident’s or their relative’s preferences and experiences? *All interviewees:* How self-evident is it that the physician knows these? And what about the pharmacist? If pharmacists would be uninformed, what would be solutions e.g., pharmacists could visit the ward to get an impression of nursing home residents. *All interviewees:* To what extent does the patient perspective need to be documented? In what way should the patient perspective be assessed? How importance is it that you/a nursing home resident know(s) why they use a medication and how they use it? How important was it that the physician was informed about your/the nursing home resident’s perspective, physical health, medical history, lab values, and allergies prior to the medication review? And what about the pharmacist? What are advantages and disadvantages of informing the pharmacist about the patient perspective and medical information? Would it be important to make a selection of nursing home residents e.g., referring patients with complex problems and not doing reviews for patients who barely use medications or not at all?
#2	*Drug reviewing* *All interviewees:* How important was the use of START/STOPP (Gallagher et al., 2008) and Beers Criteria (American Geriatrics Society 2012 Beers Criteria Update Expert Panel, 2012) (an example was given to clarify the criteria to nursing home residents/relatives and nursing staff). How useful are these criteria for clinical practice? *To physicians and pharmacists:* Was it possible to adhere to these criteria for this nursing home resident? Why or why not? Do you consider all medications for which there is a Beers or STOPP criterion inappropriate? Do the STOPP and Beers Criteria differ from each other? Is there other scientific research/knowledge through the Dutch College of Elder Care Physicians or the Dutch College of available? Were previous pharmacotherapy audit meetings useful? How important was this preparation by the pharmacist for the next step?
#3	*Multidisciplinary meeting and pharmacotherapeutic actions* *All interviewees:* How valuable was the meeting with the physician/pharmacist for this patient? Where should medication reviews be targeted at? For weighing the benefits and drawbacks of medications? For undertreatment or overtreatment? Are intermediate screenings valuable? Is the meeting a screening of problems or the best time to actually reach final decisions? What about medication errors? Would you like a nursing staff member/yourself to attend these meetings? What is important to pay attention to when it comes to one’s professional attitude?
#4	*Execution and evaluation of pharmacotherapeutic actions* *To pharmacist physician and nursing staff:* How did you agree with the physician/pharmacist about specific appointments for this nursing home resident? *To relatives/nursing home residents:* Did you hear about specific arrangements by the physician and pharmacist about your medication? *All interviewees:* How can you ensure that physicians actually provide feedback about these appointments to the pharmacy? Who is responsible for executing the changes? Which role do the nursing staff need to play during the execution? Is it important to inform or to consult family? What is the utility of asking the family? Do you inform patients about possible side effects after initiating a medication? Do you tell them about possible withdrawal effects? How does one need to organize medication reviews to obtain better results for this patient? Do the medication reviews take place with sufficient frequency? Are pharmacotherapy audit meetings useful for execution of changes?

START, screening tool to alert doctors to right treatment; STOPP, screening tool of older persons’ potentially inappropriate prescriptions.

### Qualitative Analysis

Audio-recorded interviews were transcribed verbatim by AE, a pharmacy intern. Transcripts were anonymized by replacing the participant’s name with a pseudonym (a bird name) and were subsequently entered into NVivo version 10.0 to aid data management and coding. Coding was done in an iterative fashion to allow the above described interview guide to evolve on the basis of new insights from consecutive interviews. The method of “constant comparison” was used for coding thereby expanding an initially defined coding tree with superordinate and subordinate themes. After coding of nine interviews, HW applied “coding on” by reviewing the initial codes for consistency and adequacy.

In order to reduce bias in the analysis process and to enable reflexivity, codes were regularly reviewed and discussed with co-authors AE as well as with FB, an elder care physician, and KT, a professor of clinical pharmacy. Charting was applied and rival explanations investigated to assist interpretation. Specifically, we constructed “role-ordered matrices,” one per medication review step. These were cross-tables with transcript fragments ordered according to type of interviewee i.e., nursing home resident/representative, physician, pharmacist, or nursing staff (in adjacent columns) and codes (in rows beneath each other). This enabled an examination of consensus and discrepancy among and between pharmacists, elder care physicians, nursing staff, and relatives of nursing home residents with regard to the consecutive steps of the medication reviews. For that goal, interviewees’ responses were paraphrased while maintaining a coding link to their raw statements. In multiple reviews of the data, most salient quotes were chosen. We also examined links between codes by studying overlap between them, interviewees’ reflections, and field notes. Saturation was achieved as no new themes came to the fore.

### Ethics

The medical ethical committee of the University Medical Center of Groningen approved the study (protocol number NL48091.042.14). Written informed consent was asked from nursing home residents in wards for disabling conditions if they were deemed capable by the nursing staff. Informed consent from a legal representative was asked for nursing home residents who were not capable of providing informed consent and for those from dementia special care units.

## Results

**Figure 1:**
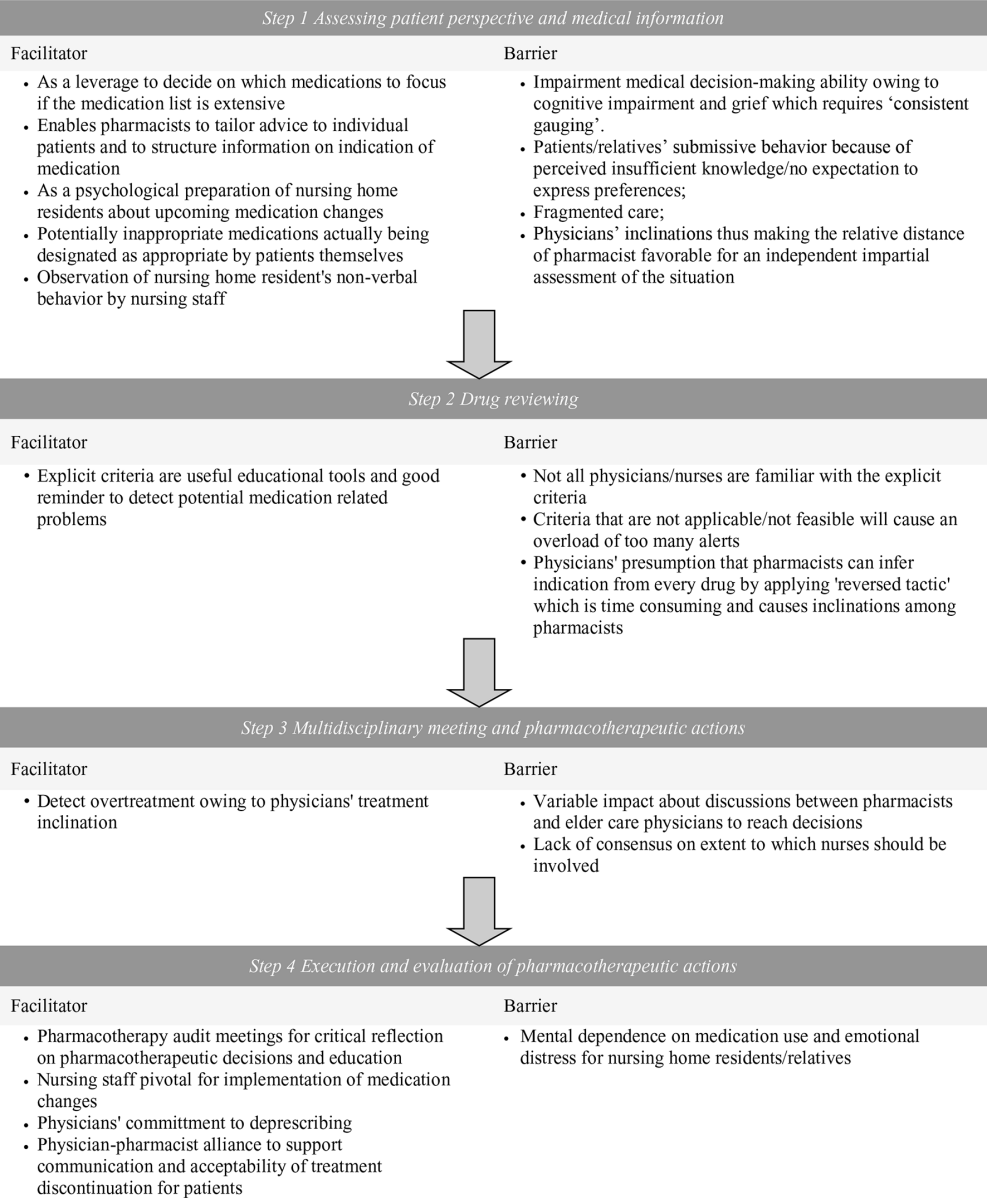
Overview of facilitators and barriers for the four 3MR medication review steps.

A total of 9 nursing home residents, 9 elder care physicians, 5 pharmacists, and 10 nurses were involved in the conduct of the 9 selected medication reviews. Of the nine nursing home residents/representatives, six participated (one representative declined the interview, one representative declined due to family circumstances, and one nursing home resident passed away). Of them, four were representatives, and two were nursing home residents. Of the nine elder care physicians, eight participated (one was unavailable during the study period). All pharmacists and nurses participated. A total of 32 semi-structured interviews were conducted. Sampling by medication review characteristics led to diversity in medications reviewed as these included psychotropic, cardiovascular, respiratory, analgesic, anti-coagulant, and bone affecting agents (**Table 2**) as well as interviewees’ characteristics (**Table 3**). In the initial phase of the analysis, the facilitators and barriers were recorded for each consecutive step of conducting medication reviews (**Figure 1**). Further analysis identified four overarching themes which described the barriers and facilitators to conducting medication reviews.

**Table 2:** Overview of nine nursing home resident cases discussed in semi-structured interviews.

1019	***Description:*** Despite the many medications prescribed, the nursing home resident (male, 75 years) is stable now. Therefore, the physician believes it undesirable to discontinue any medications. There are four medical specialists involved. Discontinuation of medications is not in line with the view of the nursing home resident’s spouse, who is highly involved in the care of the nursing home resident and who opposes discontinuation of medications. The nursing home resident is treated with amitriptyline, which is undesirable; yet, the physician believes that amitriptyline causes little harm and that an SSRI would not be sufficiently effective as an alternative. However, the pharmacist observes that the dosing is three times higher than expected, and the physician will therefore critically reappraise the dose. ***Number of medications prescribed:*** 14. ***Discussed medications:*** psycho-analeptics. ***Discontinued/dose adjusted:*** yes, dose lowering of amitriptyline. **Care unit:** for dementia
1121	***Description:*** The pharmacist recommends considering the discontinuation of statins (prescribed for high cholesterol) for this nursing home resident (female, 76 years). The physician agrees with this as statins may not be beneficial. However, in general, he finds it difficult to discontinue statins in every nursing home resident. ***Number of medications prescribed:*** 12 ***Discussed medications:*** lipid-modifying agents. ***Discontinued/dose adjusted:*** no change. **Care unit:** for dementia
1165	***Description:*** This nursing home resident (male, 67 years) has severe neuropsychiatric problems. So far, all attempts to discontinue any drug have failed. There are several medical specialists involved including a psychiatrist, a pulmonologist, and a cardiologist, who may be reluctant to discontinue medication. The psychiatrist, for instance, does not want the medications for compulsions and anxiety to be discontinued. Perhaps, this nursing home resident is too complex to discontinue medication. ***Number of medications prescribed:*** 29. ***Discussed medications:*** diuretics, corticosteroids for systemic use, thyroid therapy, mood stabilizers, antidepressants, drugs for the respiratory system. ***Discontinued/dose adjusted:*** fluvoxamine discontinued. **Care unit:** for disabling conditions
1250	***Description:*** This nursing home resident (female, 91 years) underwent a leg amputation. She experiences a lot of pain (both ischemic and osteomyelitic pain and necrotic pain) in the right foot. Of course, she does not want another amputation. Tramadol and duloxetine were prescribed for neuropathic pain after the amputation. The pharmacist recommends to reduce the dose of paracetamol that was started before duloxetine, but the physician does not believe this to be possible. The pharmacist also does not wish to discontinue duloxetine because it is difficult to differentiate between isschemic and neuropathic pain. The nursing home resident is also treated with gabapentin for neuropathic pain. Furthermore, the pharmacist recommends to discontinue statins. The cholesterol values are satisfactory, and statins may not be beneficial. The physician agrees with discontinuing statins and will discuss this with the nursing home resident. Vitamin D and calcium have not yet been started, because the physician wants to await stabilization of the nursing home resident’s condition. However, the elder care physician believes that the nursing home resident will soon pass away. ***Number of medications prescribed:*** 15. ***Discussed medications:*** analgesics, lipid-modifying agents. ***Discontinued/dose adjusted:*** lipid-modifying agents discontinued. **Care unit:** for disabling conditions
1333	***Description:*** The nursing home resident (female, 86 years) has a large amount of sleeping medications including flurazepam, oxazepam, and temazepam. However, the nursing home resident is reluctant to discontinue any of these medications. She is very anxious to sleep badly. Both the physician and the pharmacist agree that it would be a good idea to discontinue the flurazepam. However, the tapering off of this drug needs to be done in a prudent manner, because it works longer and is more lipophilic. The elder care physician says the nursing home resident is never drowsy. The pharmacist still warns that there is an increased fall risk as this nursing home resident often uses a wheel chair to move around. The elder care physician asks advice on whether and how to switch from flurazepam to an alternative drug in case he decides to taper this drug. The pharmacist will provide detailed advice. As a start, the pharmacist believes it is best to first taper off medications to lower dosages. ***Number of medications prescribed:*** 18. ***Discussed medications:*** anxiolytics. ***Discontinued/dose adjusted:*** flurazepam discontinued. **Care unit:** for disabling conditions
1360	***Description:*** This nursing home resident (female, 96 years) is treated with venlafaxine. The pharmacist asks if there is an indication for this drug, and whether the nursing home resident has depression. The elder care physician confirms depression but thinks that, since the patient has moved to a nursing home, much of the previously experienced distress may have disappeared, thereby making venlafaxine redundant. The pharmacist wonders whether paracetamol is still needed. The physician confirms the necessity of paracetamol as the nursing home resident has painful legs. Finally, the pharmacist asks if vitamin D and bisphosphonates should be initiated. However, the physician does not believe this to be useful, since the nursing home resident is not mobile. ***Number of medications prescribed:*** 5. ***Discussed medications:*** antidepressants, medications for bone diseases. ***Discontinued/dose adjusted:*** no change. **Care unit:** for dementia
1415	***Description:*** This nursing home resident (male, 91 years) has had a stroke, has edema, pain, a dry mouth, and a pulmonary embolism. The nursing home resident was initially treated with acenocoumarol because of atrial fibrillation. However, owing to fall risk and increased risk of bleedings the nursing home resident was switched to acetylsalicylic acid and dipyridamole. The patient is still somewhat mobile and can walk. Owing to the nursing home resident’s age and decline, re-initiating acenocoumarol is no longer indicated. Omeprazole is prescribed as a gastroprotective agent because of the prescribing of acetylsalicylic acid. Paracetamol may be discontinued. ***Number of medications prescribed:*** 8. ***Discussed medications:*** antithrombotic agents, diuretics, medications for peptic ulcer, and gastro-oesophageal reflux disease. ***Discontinued/dose adjusted:*** this patient became deceased before he or his family could be interviewed. **Care unit:** for disabling conditions
1439	***Description:*** This nursing home resident (female, 79 years) uses no medications at all. She has dementia, but she is vital, and her lab values are satisfactory. ***Number of medications prescribed:*** 0. ***Discussed medications:*** not applicable. ***Discontinued/dose adjusted:*** not applicable. **Care unit:** for dementia
1823	***Description:*** This nursing home resident (female, 88 years) has a history of orthostatic hypotension, hypertension, stroke, and Bowen’s disease. Lab values including thyroid gland function were normal. The nursing home resident is also treated with paracetamol for back pain and macrogol to improve sleeping. She has a blood pressure of 130/72. She is treated with spironolactone and a thiazide. The physician finds it difficult to comprehend this treatment combination of spironolactone and thiazide. The physician will clarify the rationale for this treatment combination and determine the blood pressure again. ***Number of medications prescribed:*** 10. ***Discussed medications:*** antithrombotic agents, diuretics. ***Discontinued/dose adjusted:*** spironolactone and thiazide discontinued. **Care unit:** for disabling conditions

**Table 3:** Characteristics of nursing home residents, elder care physicians, pharmacists, and nurses involved in the nine selected medication reviews.

Statistic	Residents/representatives	Pharmacists	Physicians	Nursing staff
N	6 *	5 **	8 ^§^	10 ^†^
Age, M (SD)	78 (10)	42 (12)	46 (13)	43 (10)
Female, n	4	2	2	9
No. of prescribed medications, median (range)	12 (8)	− − ^‡^	− − ^‡^	− − ^‡^
>10 years of working experience, n	− − ^‡^	3	4	9

*Of the 9 nursing home residents/representatives, 6 agreed to participate, 1 representative declined the interview, 1 representative declined due to family circumstances and 1 resident passed away; 4 were representatives and 2 were nursing home residents.

**1 pharmacist conducted 3 medication reviews, 2 pharmacist conducted 2 medication reviews each, and 2 pharmacists conducted 1 medication review each.

^§^Of the 9 elder care physicians, 8 participated, 1 was unavailable during the study period.

^†^1 additional member of the nursing staff was involved in the interview about the medication review of case 1823.

^‡^Not applicable.

### Theme 1: Realizing Fidelity of the Patient Perspective

In step 1 of the medication review, it became clear that realizing fidelity of the patient perspective in the medication review process (i.e., correctly assessing and incorporating nursing home residents’ or relatives’ viewpoints) proved to be a delicate balance between facilitators and barriers. The facilitators included the utility of the patient perspective as a leverage to decide on which medications to focus on in cases where an extensive number of medications were prescribed, for tailoring the pharmacist’s advice to the physician, and to psychologically prepare nursing home residents or their relatives for medication changes. Importantly, there could be discordant views on medications, with clinical guidelines designating medications as inappropriate, and candidates for discontinuation, while patients considered them to be needed:

“The question is whether these medications are inappropriate for this nursing home resident. He has end-stage COPD and has already lived longer than expected. He also makes a deliberate choice to have the least amount of pain and bother. I would not wish this combination of medications on anybody.” *[Swan, physician discussing case 1165)*

Important barriers to involving the patient became evident as well. As expected, a barrier to acquiring a reliable understanding of the nursing home resident’s perspective was their cognitive impairment. In such cases, an important strategy for physicians was to apply consistent gauging to assess the nursing home resident’s viewpoint:

“You always try to verify nursing home residents’ answers. By checking the response you received and the consistency between answers after repeating your question … But also by coming back to repeat the same conversation, or verifying your opinion with the family or the nursing staff …” *[Cormorant, physician discussing case 1250]*

Grief was also a barrier to involving the nursing home resident’s or their family’s perspective. This came to the fore in an interview with a spouse of a recently admitted nursing home resident:

I am having a very difficult time with this. She does not seem to be my wife anymore. I do not want her to die … please understand me correctly … but it makes a difference. I cannot judge her joy in life … but from my point of view, living in a nursing home would be a nightmare. *[Buzzard, spouse discussing case 1439]*

It was also exemplified by the following statement from a member of the nursing staff in relation to a nursing home resident who experienced the loss of a limb:

“We are very careful with this nursing home resident … You always ask her what she wants and what she still can do. This woman is also going through the whole process of accepting [her leg amputation], which also confuses her entirely. What a misery”. *[Eagle, nurse discussing case 1250]*

The submissive behavior of some nursing home residents/representatives could also be a barrier hampering healthcare professionals’ understanding of the patient perspective. Nursing home residents/representatives often did not expect to be involved in the medication review process and preferred to trust their physician’s advice:

“I don’t think my mother has ‘preferences.’ She has always been very compliant in swallowing what was prescribed to her. Perhaps because of too little knowledge. Perhaps it is the generation who simply does what the doctor prescribes without questioning.” *[Duck, daughter of nursing home resident discussing case 1250]*

This expectation was possibly related to a lack of knowledge as confirmed by another representative:

“My mother sometimes expresses reluctance to take a medication, but she does not know what the medication is prescribed for and she cannot appraise this. What do I need to imagine when it comes to preferences? Suppose, I would apply this to myself. I also have several medications. Preference for one medication over another requires you to be informed about your medications, which I am not.” *[Crane bird, son of nursing home resident discussing case 1360]*.

Establishing the nursing home resident’s perspective was mainly seen as the role of the physician, but fragmented care, particularly less regular contact with nursing home residents, was perceived as a barrier to the physicians knowing the patient perspective well enough:

“I do not exactly know about every nursing home resident’s preferences. This has to do with the presence of many other physicians and that you do not see the nursing home residents as often as you used to see them”. *(Raven, physician about case 1121)*

### Theme 2: Level of Comprehensiveness of Medication Reviews

In step 2 of the medication review, the “level of comprehensiveness of medication reviews” was an important theme that concerned the need to find an optimum between comprehensiveness and feasibility. Pharmacists considered published tools such as the START (screening tool to alert doctors to right treatment), STOPP (screening tool of older persons’ potentially inappropriate prescriptions) (Gallagher et al., 2008), and Beers Criteria (American Geriatrics Society 2012 Beers Criteria Update Expert Panel, 2012) as facilitators of medication reviews. Pharmacists described them as useful educational tools and good reminders to detect potential medication-related problems. A barrier, however, for physicians and nursing staff was the lack of their familiarity with these tools. Moreover, physicians explained that some of these tools may use an overly comprehensive set of criteria which tended to result in an overload of potentially inappropriate medication alerts:

“If you strictly adhere to the Beers criteria, you will get many alerts. In the past we discussed some of the alerts with the pharmacists and requested them to omit these alerts, as we did nothing with them.” *[Swan, physician discussing case 1165]*

This was confirmed by a pharmacist who knew from experience that recommendations from these tools could not always be implemented in practice:

“I have done so many reviews. …. And you know indeed that you should discontinue temazepam, but in practice you know it will not succeed. Well in your head, you notice them, but you do not always act according to these criteria.” *[Magpie, pharmacist discussing case 1823]*

Related to this was the need to also consider the adaptation of guidelines to individual patients as acknowledged by one of the representatives:

“Yes … it is excellent that there are guidelines for medications and to administer medications at an optimal dose. At the same time, adaption of a dose to the individual person is of utmost importance as well. Setting the dose of haloperidol according to the guidelines suited the needs of m+y partner well and she thrived on it as there is a stable situation now. However, I also heard a story of an 85-year old resident who was prescribed haloperidol for the first time and could not get out of bed anymore” [Hummingbird, spouse discussing case 1121].

### Theme 3: Inclinations of Healthcare Providers

In steps 1–3 of the medication review, inclinations i.e., usual practices and habits of physicians, pharmacists, and nursing staff were observed. Pharmacists seemed to lack confidence in making judgments about the patient perspective because they felt they were too far removed from the patients. While this seemed to be a barrier at first glance, some physicians actually saw this relative distance as a facilitator, since it allowed the pharmacist to assess the situation more impartially and to proactively counteract physicians’ inclinations:

“It may be a good idea to let someone, who does not know anything about the situation [such as the pharmacist], critically appraise the medication use and patterns and to let them learn only afterwards about the reasons for certain choices.” *[Cormorant, physician discussing case 1250]*

Different inclinations were also observed with respect to knowledge of nursing home residents’ medical history. During the DIM-NHR study, pharmacists had routine access to nursing home residents’ medical charts, which is uncommon in usual care. Pharmacists highly valued receiving this medical information as part of the trial as it served as a facilitator to conducting medication reviews. They felt they were better able to tailor their advice to the individual patient; it saved time, and they were less liable to their own inclination of inferring diagnostic indications from medications:

“To me having the medical history on paper is of utmost importance. It really saves a lot of discussion time with the physician. Otherwise, I tend to ask questions, because you adopt a reversed tactic and you try to infer the indication from the medication (…).” *[Bittern, pharmacist discussing case 1250]*

This valuing by pharmacists was confirmed by the representatives of nursing home residents:

“It is not necessary for pharmacists to know everything, but it would be important to know what is wrong with someone, what kind of medications are prescribed for their conditions and whether everything connects with anything. If they would know this, this would earlier sound the alarm bell, because a physician does not know this *[Duck, daughter of nursing home resident discussing case 1250]*.”

In contrast, a potential barrier for pharmacists was that physicians thought that pharmacists should infer the indication from the medication and need not have access to nursing home residents’ medical charts:

“A pharmacist talks from a different angle and they should not sit on our chairs in my opinion. … We have more profound clinical knowledge. We just expect them to judge whether combinations of medications we prescribe are possible. And we are the ones who study the nursing home resident’s diseases, while the pharmacist infers diseases from the nursing home residents’ medication use.” *[Swan, physician discussing case 1165]*

Inclinations of physicians bared important implications for setting intervention targets of medication reviews. Efficacious targets were preferably complementary to the usual practices of physicians. Physicians considered medication reviews particularly useful for reducing overtreatment rather than undertreatment owing to their tendency toward starting rather than stopping medications:

“Physicians tend to put more emphasis on undertreatment than on overtreatment. Starting medication is perceived by physicians as something active whereas discontinuing medication is not. That is an inclination I think. Of course, you know you also have to do something about overtreatment but when you are rushing around, it intuitively feels more problematic when you do not start a medication rather than forgetting to discontinue another one.” *[Raven, physician discussing case 1121]*

Another reason why medication reviews were considered useful for reducing overtreatment may be explained by a view among physicians that undertreatment is less clinically relevant than overtreatment:

“What chance is there that nursing home residents experience a medical problem as a result of undertreatment, because their life expectancy is so short?… This is what I use medication reviews for: Which blood pressure medications do people have? If there are three, I give it a sharp look to consider whether a drug can be discontinued.” *[Pheasant, physician discussing case 1360]*

Sometimes, medication changes were executed immediately. In other cases, decisions were postponed. A barrier observed by one pharmacist that particularly related to the case of postponed decisions was the seemingly variable impact or utility of their discussions with elder care physicians:

“There are different physicians, and different kinds of discussions. For some physicians you notice that they literally adjust the drug prescriptions behind their computers and you see that they follow your advice, whereas other physicians nod a few times and make notes in a notebook (…).” *[Stork, pharmacist discussing case 1019]*

A clear barrier was the lack of consensus on the extent to which nurses should also join the medication review meetings between pharmacists and physicians. One pharmacist thought nurses lacked pharmacotherapeutic knowledge and foresaw emotional involvement with patients as an undesirable inclination of nurses:

“You have to keep the medication review as clear as possible and you will also get more emotional input, rather than pharmacotherapeutic knowledge, because the nursing staff does not possess this knowledge.” *[Bittern, pharmacist discussing case 1250]*

However, another pharmacist thought that the information held by nursing staff was valuable for medication reviews:

“Well often initiation of medications is discussed [without the nurse being present]. But then, you do not know precisely if a patient has constipation, or difficulties with taking their medications. The physician does not always know this … The nursing staff does.” *[Pelican, pharmacist discussing case 1439]*

Nursing staff, however, thought it too time consuming to participate in the medication review discussions, which would be at the expense of their caring duties.

### Theme 4: Inter-Professional Collaboration and Alliances

In steps 1 and 4 of the medication review, the importance of “interprofessional collaboration and alliances” between physicians, pharmacists, and nurses was demonstrated. Physicians valued pharmacotherapy audit meetings with pharmacists held once every 6 weeks but which were not part of the 3MR. These meetings, in which physicians and pharmacists critically reflected on pharmacotherapeutic decisions and educated each other, were considered a facilitator for conducting medication reviews.

In the physician–nurse alliance, both nurses and physicians indicated that the key role of the nurses was to facilitate the implementation of medication changes. Nursing staff assisted physicians in signaling possible relapse and other problems, as well as communicating the medication changes with nursing home residents and/or their relatives by translating physicians’ jargon into lay language. This was confirmed by nursing home residents and representatives. For example, where the nurse was able to convey the physician’s commitment to deprescribing to the family, they would be more likely to agree with medication changes:

“Yes if the physician has a clear vision on stopping medication or tapering these, the family will follow their path. If the physician also considers it of utmost importance to be very critical about the medication, then that makes a difference of course.” *[Canary, nurse discussing case 1121]*

Beyond this, nurses felt they had additional roles to play in order to support elder care physicians with medication reviews. In circumstances in which the patient perspective would be difficult to assess, a facilitator offered by nurses was to carefully observe the nursing home residents’ behavior to verify the nature of the problem:

“The things that a nursing home resident [or his representative] tells you about are not always the things that bother them. (…) for example, in this case the resident’s spouse reports that he is in pain. However, we observe restlessness. … It is important to distinguish between the two.” *[Parakeet, nurse discussing case 1019]*

The importance of good inter-professional alliances was underlined by the observed barrier that physicians and nursing staff found it sometimes challenging to communicate discontinuation of medications with nursing home residents or their representative owing to the nursing home residents’ mental dependence on medications or associated emotional distress:

“She has fixed customs and has difficulty to accept change. So despite the insult to her memory function, there is always the possibility that when you discuss medication changes with her, she will become anxious about it, about what might happen to her.” *[Pheasant, physician discussing case 1360]*

Indeed, a physician suggested the formation of a physician–pharmacist alliance to better support communication and acceptability of treatment discontinuation. If a pharmacist would show concordance with the physician’s decision-making, this may increase a nursing home resident’s or their relative’s confidence in review decisions:

“Suppose that people understand everything very well and that I have difficulty to convince them. Then it could be convenient to have the pharmacist at my side. In that case, I could say: would you like to join me some time? I have a critical patient with quite some preferences or specific drug related problems.” *[Myna, physician discussing case 1333)*

This was acknowledged by one of the representatives adopting a physician’s perspective while explaining his point of view:

“It would give additional authority to a decision. This decision is not only taken by me but also by the pharmacist. That sort of thing could carry a bit more weight.” *[Crane bird, son of nursing home resident discussing case 1360]*.

## Discussion

This qualitative analysis of conducting medication reviews in the nursing home setting provided important insights into facilitators and barriers of patient involvement and inter-professional collaboration needed for conducting the consecutive steps of medication reviews. The facilitators and barriers observed in this study may help explain why findings about the effectiveness of medication reviews in reducing inappropriate prescribing were equivocal (Alldred et al., 2016) and may provide guidance for future improvement.

### Theme 1: Realizing Fidelity of the Patient Perspective

With respect to incorporating the patient perspective in medication reviews, *realizing fidelity of the patient perspective* was found to be a delicate balance between a number of facilitators and barriers. Perceived facilitators of the patient perspective were an improved focus on which medications to target for change and also preparing patients psychologically for treatment adjustments. A notable observation here was that overprescribing could be partly guided by a nursing home resident’s values. This observation underlines the need to incorporate shared decision-making in order to weigh the ethical principle of “patients’ autonomy” against the other ethical principles of “beneficence,” “non-maleficence,” and “justice” when physicians and pharmacists appraise the appropriateness of medications. However, barriers in assessing the patient perspective were also evident. As expected, the patient perspective was often difficult to ascertain, particularly for patients with dementia or cognitive impairment. In these cases, a reported facilitator was the application of consistent gauging to verify the consistency of the patient perspective. Furthermore, the importance of adopting meta-communication, reflection, and expression of mutual expectations with nursing home residents and/or relatives was also highlighted for two circumstances. First, in addition to cognitive impairment, grief was found to be a barrier to involving the nursing home resident in medication reviews. Thus healthcare providers must come to appreciate that although nursing home residents may be *eligible* according to guidelines for a medication review, it would also be important to verify if they themselves are ready for it. Second, our findings demonstrated that patients may feel they lack sufficient knowledge to communicate their preferences in medication reviews. Physicians should therefore emphasize the importance of nursing home residents’ (or their relatives) lay input to increase their confidence.

Some of our findings on patient involvement mirror those of previous studies. Inappropriate prescribing was found to depend in part on whether patients perceived their own medications as inappropriate (Reeve et al., 2013; Reeve et al., 2017). As one would expect, establishing patient preferences was found to be challenging in nursing home residents with cognitive impairment (Reeve et al., 2016). But in the present study, we also observed this to be difficult in case of grief. The consistent gauging strategy which came to the fore in this study seems to correspond with verification in an ongoing dialogue (Reeve et al., 2016). Our finding that nursing home residents were “submissive” or tended to rely on their physician’s judgment is consistent with paternalistic decision-making (Spinewine et al., 2005; Hughes and Goldie, 2009) but, in this study, seemed mostly to originate from a lack of knowledge about medications among nursing home residents/relatives. For conducting meta-communication dialogues and improving shared decision-making between physicians and nursing home residents, a previously proposed framework for shared decision-making with regard to deprescribing could be helpful (Jansen et al., 2016).

### Theme 2: Level of Comprehensiveness of Medication Reviews

With respect to the *comprehensiveness of medication reviews*, it emerged that an optimum between comprehensiveness and feasibility was important given the likelihood that a redundancy of criteria of inappropriate prescribing would cause an overload of alerts for clinicians. A proactive strategy would therefore be to discuss beforehand whether a selection and or refinement of START/STOPP and Beers Criteria would be appropriate. These recommendations complement previous related findings showing that physicians thought START/STOPP (Gallagher et al., 2008) and Beers Criteria for medication analysis (American Geriatrics Society 2012 Beers Criteria Update Expert Panel, 2012) to be helpful but lacking evidence (Cornege-Blokland et al., 2012).

### Theme 3: Inclinations of Healthcare Providers

There were a number of distinct *inclinations of physicians, pharmacists, and nursing staff* observed in this study. While pharmacists saw their relative distance from the patient perspective as a disadvantage, physicians saw pharmacists’ impartialness as beneficial in order to compensate for their own inclinations and background knowledge of patients. However, being at a distance from the medical history was a barrier for pharmacists leaving them to infer indications from medication prescriptions. Thus, physicians and pharmacists need to decide together where distance should be eliminated and where it should be maintained to optimize the medication review process. A related observation was that physicians were not always able to assess the patient perspective due to high workload and interruptions, as has been previously noted (Cullinan et al., 2015). This would support the observation that nursing staff can provide a greater role than mere implementation of treatment changes.

Furthermore, to increase the feasibility of reviews, the intervention targets of medication reviews should be complementary to physicians’ usual practice. Physicians awareness of their tendency to start medications rather than stop them may highlight the need to focus on overtreatment as a primary intervention target of medication reviews. This qualitative observation may actually explain the quantitative findings from the DIM-NHR study showing that 3MR medication reviews were effective in discontinuing inappropriate medication use, but not in initiating potentially underprescribed medications (Wouters et al., 2017).

### Theme 4: Inter-Professional Collaboration and Alliances

While alliances between physicians and pharmacists seemed firmly established with respect to medication reviews, our findings indicated a lack of consensus between healthcare providers on the involvement of nurses. This requires attention, especially given the nurses’ potential contribution described by interviewees such as careful observation and insight into the patients’ perspective and lay translation of medical information which may facilitate the acceptability of treatment changes by nursing home residents/relatives. Pharmacists could further substantiate physicians’ treatment change decisions, especially in cases where nursing home residents have become emotionally dependent on medications. This mental dependence seems to relate to the persistent difficulty of deprescribing being interpreted by nursing home residents as an implicit sign that they are being given up on (Schuling et al., 2012). Altogether, our findings underline the importance to also adopt meta-communication between healthcare providers. This is likely to foster a discussion about arrangements, the specificities of the processes, mutual expectations, and predefined agreements. This may prevent inertia (Reeve et al., 2017) and devolving responsibility or passing the responsibility from one healthcare professional to another (Kouladjian et al., 2016). To that end, our findings also suggested to embed medication reviews in existing pharmacotherapy audit meetings of physicians and pharmacists which are held to agree on prescribing guidelines and to discuss other pharmacotherapeutic issues.

### Methodological Considerations

A number of methodological issues need to be addressed. Specific strengths of our study included the purposive sampling of salient medication reviews which was supported by the range of background characteristics including a variety of medical problems and types of medications, and the number of prescribed medications including one patient who used no medications at all. A related strength was that we interviewed all relevant stakeholders, i.e., nursing home residents (or their relatives), elder care physicians, pharmacists, and nursing staff. Thus, purposive sampling of medication reviews and conducting the interviews with these stakeholders may have contributed to the breadth of barriers and facilitators as well as the identification of novel ones. Lastly, the rigorous reviewing of the codes as well as applying charting and “role ordered matrices” is likely to sustain that the present findings reflected key barriers and facilitators.

Possible limitations include the following. We could only include a relatively small number of nursing home residents/representatives. We did not ask direct questions about barriers as this may have resulted in stating the obvious, e.g., complaining about work load, which would distract the physician/pharmacist from talking about more subtle clinical problems. Even more important was that we needed to be prudent. At the time of this research, there was much controversy about inappropriate prescribing in the media. Asking directly about barriers would have caused misinterpretation and frustration among clinicians and nursing staff. The fact that some pharmacists were involved in more than one review may also have given them a broader perspective than other pharmacists interviewed. Further, given their lesser involvement in medication reviews, nursing home residents and nursing staff may have perceived interview questions differently than pharmacists and physicians making mutual comparisons less straightforward. Lastly, we cannot rule out social desirability although healthcare professionals were interviewed confidentially and were open to discussing barriers.

## Conclusion

Taken together, four key themes of salient barriers and facilitators of conducting medication reviews were identified. These require the need to improve meta-communication during the medication review process. This pertains to the need for healthcare providers to appraise the fidelity of the patient perspective in a dialogue with nursing home residents/relatives. Furthermore, discourse between healthcare practitioners is needed beforehand about the comprehensiveness and intervention targets of medication reviews as well as inter-professional collaboration. In turn, this has the potential to improve effectiveness of medication reviews aimed at the deprescribing of inappropriate medications in nursing home residents.

## Data Availability

Data are available through KT: k.taxis@rug.nl.

## Ethics Statement

The study involving human participants was reviewed and approved by University Medical Center of Groningen. The patients/participants provided their written informed consent to participate in this study.

## Author Contributions

Study concept and design: HW, FB, KT, and SZ; Acquisition of participants: HW, AE, SZ, and KT; Data analysis: HW, AE, FB, and KT; Interpretation of data: HW, JF, AE, LO, SZ, FB, KT; and Preparation of manuscript: HW, JF, LO, SZ, FB, and KT.

## Funding

This work was supported by a grant from the Netherlands Organization for Health Research and Development (grant number: 80-83600-98-10176).

## Conflict of Interest Statement

The authors declare that the research was conducted in the absence of any commercial or financial relationships that could be construed as a potential conflict of interest.
